# Influence of genetically predicted autoimmune diseases on NAFLD

**DOI:** 10.3389/fimmu.2023.1229570

**Published:** 2023-09-11

**Authors:** Min Xu, Tong Wu, Zhaoxia Li, Guijie Xin

**Affiliations:** Department of Hepatology, The First Hospital of Jilin University, Changchun, China

**Keywords:** Mendelian randomization (MR), autoimmune disease, non-alcoholic fatty liver disease (NAFLD), causality, gut microbiota, risk factor

## Abstract

**Introduction:**

Non-alcoholic fatty liver disease (NAFLD), the emerging cause of end-stage liver disease, is the most common liver disease. Determining the independent risk factors of NAFLD and patients who need more monitoring is important.

**Methods:**

Two-Sample Mendelian randomization (MR) was performed in the analysis to investigate the causal association of different autoimmune diseases with NAFLD using summary level data. Genome-wide association study (GWAS) of 5 autoimmune diseases including celiac disease (CeD), Crohn’s disease (CD), multiple sclerosis (MS), rheumatoid arthritis (RA), and type 1 diabetes (T1D) were selected for Instrument variables (IVs). NAFLD was included as outcome.

**Result:**

After adjusting for confounding factors, genetic predisposition of CeD (OR= 0.973, [0.949,0.997], IVW p-value=0.026), MS (OR= 1.048, [1.012,1.085], IVW p-value= 0.008), RA (OR= 1.036, [1.006,1.066], IVW p-value=0.019), T1D (OR= 1.039, [1.002,1.079], IVW p-value= 0.041) is causally associated with NAFLD. No causal effect was found between CD and NAFLD.

**Conclusion:**

CeD itself may be a protective factor for NAFLD, the results of previous observational studies have been influenced by confounding factors, and the morbidity of NAFLD may be higher in patients with MS, RA, and T1D than in common populations, and monitoring the prevalence of NAFLD in these populations is considerable.

## 1 Introduction

Non-alcoholic fatty liver disease (NAFLD) is the most common chronic liver disease in the world (1), with a prevalence of close to 25% in the general population, causally associated with obesity and type 2 diabetes (T2D) (2). NAFLD includes a spectrum of liver pathologies including simple steatosis, steatohepatitis, fibrosis, and cirrhosis. Due to its high prevalence, NAFLD is currently the fastest-growing cause of liver-related death worldwide and is emerging as a significant cause of end-stage liver disease, primary liver cancer, liver transplantation, and a significant health economic burden (3). Therefore, it is important to determine the independent risk factors for NAFLD and which patients should receive more stringent health monitoring.

Autoimmune diseases occur when the immune response is misdirected to the tissues of the self, ultimately leading to structural and functional disorders of the body and organs. Genetic factors, environmental influence, and immune abnormalities (including infections) often combine to trigger autoimmune diseases (4). There are nearly 100 identified autoimmune diseases, the most common of which include systemic lupus erythematosus (SLE), rheumatoid arthritis (RA), multiple sclerosis (MS), type 1 diabetes (T1D), Graves’ disease (GD), inflammatory bowel disease (IBD), etc. (5). Considerable epidemiological evidence links these disorders to NAFLD, and most are tend to increasing risk of increased NAFLD (6–10).

The gold standard for exploring causality is randomized controlled trials. In theory, randomized controlled trials require strict randomization of patients with different autoimmune diseases, intervention, and follow-up to document the incidence of NAFLD. Such trials are difficult to conduct because of the time and high cost required. Mendelian randomization (MR) is a statistical research method similar to randomized controlled trials, where MR uses a genetic variation to determine whether the observed association between exposure and outcome is consistent with a causal effect (11). MR relies on the natural random combination of genetic variants during meiosis, resulting in a random distribution of genetic variants in the population (11), and is not prone to confounding factors and reverse causal associations.

In this study, MR was used to investigate the causal relationship between several autoimmune diseases and NAFLD.

## 2 Methods

### 2.1 Two-sample MR study design

Based on summary-level data, two-sample MR was performed to investigate the causal association between different autoimmune diseases and NAFLD. A total of 5 autoimmune diseases including celiac disease (CeD), Crohn’s disease (CD), multiple sclerosis (MS), rheumatoid arthritis (RA), and type 1 diabetes (T1D) were selected for our MR study (12–18), **Table 1** provides detailed information on all the summary data used in the study.

**Table 1:** Detailed information of data used for study.

Phenotype	Ancestry	Cases/Controls	nSNP	PMID
Celiac Disease	European	12,041/12,228	38	22057235
Crohn’s Disease	European	17,897/33,977	130	26192919
Multiple Sclerosis	European	14,498/24,091	47	24076602
Rheumatoid Arthritis	European	5,539/20,169	10	20453842
Type 1 Diabetes	European	9,266/15,574	47	32005708
NAFLD	European	8,434/770,180	–	34841290

nSNP, the number of single nucleotide polymorphism; PMID, ID of publication in the PubMed; NAFLD, Nonalcoholic fatty liver disease.

**Figure 1:**
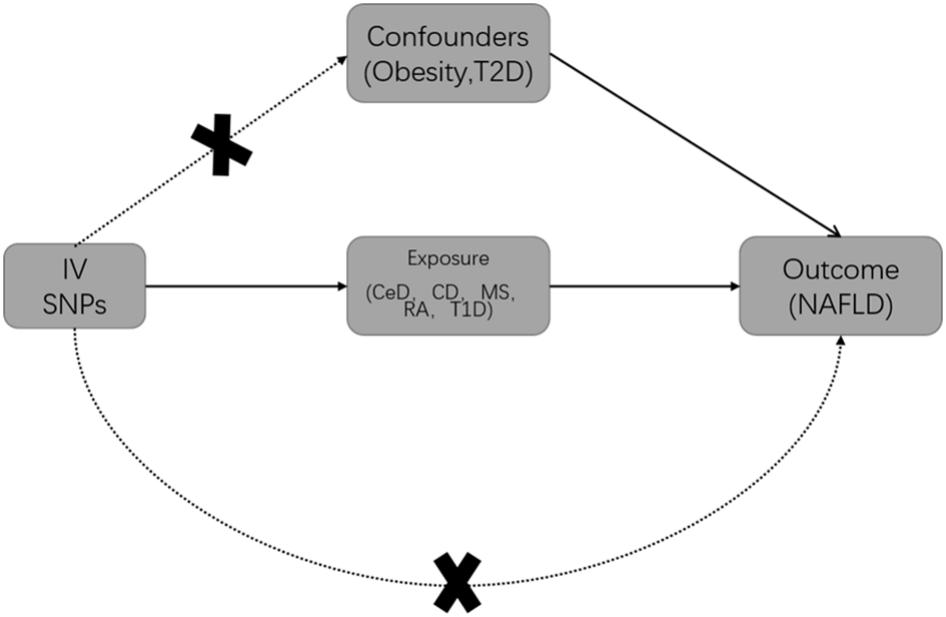
Basic Mendelian randomization (MR) framework for investigating the causal relationship between autoimmune diseases and NAFLD. T2D is Type 2 diabetes; IV is instrumental variable; CeD is celiac disease; CD is Crohn’s disease; MS is multiple sclerosis; RA is rheumatoid arthritis; T1D is Type 1 diabetes; NAFLD is non-alcoholic fatty liver disease.

MR studies should be conducted under three main assumptions (19): Genetic variables should be closely related to exposure (12–16); Genetic variables should not be associated with any confounders that may affect exposure and outcome; Genetic variables can only influence outcomes through exposure. First of all, Qualified IVs were selected based on strict selection criteria. Genetic variants were selected as IVs based on their genome-wide significance (GWAS p-value < 5×10−8) and linkage disequilibrium. (LD, r2 = 0.001, clump window 5000 kilobases). For the selection of qualified IVs, only summary-level GWAS data from European ancestry were utilized. Secondly, SNPs (Single Nucleotide Polymorphism) associated with obesity and T2D were excluded from exposure by Phenoscanner (PhenoScanner (cam.ac.uk)). It is known that obesity and type 2 diabetes are risk factors for NAFLD (3), Inclusion of these SNPs would violate the second and third assumptions (20) (**Figure 1**). Additionally, exposure and outcome data were harmonized to ensure that each IV had the same effect allele. Finally, MR analysis was performed on the eligible SNPs.

### 2.2 Data source for autoimmune diseases and NAFLD

#### 2.2.1 Celiac disease

The IEU Open GWAS project (mrcieu.ac.uk) provided summary-level data for CeD (12), which included 12,041 cases and 12,228 controls, identifying 38 SNPs associated with CeD. A total of 35 exposure-related SNPs were found in the outcome, 3 SNPs associated with potential confounders (rs3184504, rs6457617, rs13195040), and no SNPs were excluded through harmonization.

#### 2.2.2 Crohn’s disease

The IEU Open GWAS project (mrcieu.ac.uk) provided summary-level data for CD (13), which included 17,897 cases and 33,977 controls, identifying 130 SNPs associated with CD. A total of 122 exposure-related SNPs were found in the outcome, and 12 SNPs associated with potential confounders (rs13109404, rs13407913, rs17391694, rs2641348, rs2641348, rs26528, rs3184504, rs3197999, rs56163845, rs6908425, rs77981966, rs780094, rs9264942) and 7 SNPs palindromic with intermediate allele frequencies (rs10878302, rs10956252, rs10995271, rs1927681, rs2266961, rs2847293, rs35730213) were excluded.

#### 2.2.3 Multiple sclerosis

The IEU Open GWAS project (mrcieu.ac.uk) provided summary-level data for MS (14), which included 14,498 cases and 24,091 controls, identifying 47 SNPs associated with MS. A total of 45 exposure-related SNPs were found in the outcome, including two potential confounders (rs11554159, rs3131283), and no SNPs were excluded through harmonization.

#### 2.2.4 Rheumatoid arthritis

The IEU Open GWAS project (mrcieu.ac.uk) provided summary-level data for RA (mrcieu.ac.uk) (15). The GWAS included 5,539 cases and 20,169 controls, identifying 10 SNPs associated with RA. All of the exposure-related SNPs were found in the outcome, one SNP associated with potential confounders (rs497239), and no SNPs were excluded through harmonization.

#### 2.2.5 Type 1 diabetes

The IEU Open GWAS project (mrcieu.ac.uk) provided summary-level data for T1D (16). The GWAS included 9,266 cases and 15,574 controls, identifying 47 SNPs associated with T1D. A total of 33 exposure-related SNPs were found in the outcome, one SNP associated with potential confounders (rs8056814) and 6 SNPs palindromic with intermediate allele frequencies (rs10865468, rs17125653, rs34296259, rs34536443, rs55996894, rs689) were excluded.

#### 2.2.6 NAFLD

Our study mainly focused on the association between CeD, CD, MS, RA, and T1D. For the outcome data, we chose recent GWAS summary data to date. The sources of datasets are listed in **Table 1**.

Summary-level data for NAFLD were acquired from the GWAS Catalog (https://www.ebi.ac.uk/gwas/home), they performed 2 new GWASs in the UK Biobank and Estonian Biobank and performed a meta-analysis of 4 cohorts (UK Biobank, Estonian Biobank, eMERGE, and FinnGen), totaling 8,434 NAFLD cases, all identified through EHRs (electronic health records), with 770,180 controls (17). All the GWAS have been approved by corresponding Ethics Committees.

### 2.3 Data reliability analysis

As mentioned above, obesity and T2D are important risk factors for NAFLD (3), and epidemiological and animal experimental evidence for these conclusions has been validated in previous MR analyses (21), and both factors were excluded as confounders in our IVs, so we selected these two factors to validate the reliability of the NAFLD database.

Summary data for BMI, including 461,460 European ancestry individuals, were obtained from the consortium of the MRC Integrative Epidemiology Unit (MRC-IEU) (GWAS ID: ukb-b-19953), and summary data of T2D were retrieved from the Meta-analysis of three GWAS datasets of European ancestry in a very large sample of T2D (62,892 cases and 596,424 controls) (22).

Multiple testing was controlled by the Benjamini-Hochberg method. Results with an adjusted FDR less than 0.05 were considered to be significantly causally related. In addition, results at a threshold of FDR less than 0.1 were considered to imply an significant association.

### 2.4 Statistic analysis and data visualization

To estimate the combined effect of all SNPs, the inverse-variance-weighted (IVW) method was performed (23). Additionally, as complementary MR methods, weighted median (24), MR-Egger regression (25), simple mode, and weighted mode (26), were performed.

As a result of differences in analysis platforms, experimental conditions, inclusion populations, and SNPs, two-sample MR analysis may have heterogeneity. Therefore, the Cochrane’s Q test was performed to appraise heterogeneity, if the P-value > 0.05, it is considered that there is no heterogeneity in the IVs, and the influence of heterogeneity on the estimation of causal effects can be ignored (27). Forest plots visualized the heterogeneity (28)., MR-Egger regression analysis was used to calculate the intercept and P value of the sensitive SNPs of horizontal pleiotropy.

Statistic analysis and data visualization were performed using TwoSampleMR package (version 0.5.6) (23) and MR-PRESSO package (29) in R (version 4.2.2).

## 3 Result

**Figure 2:**
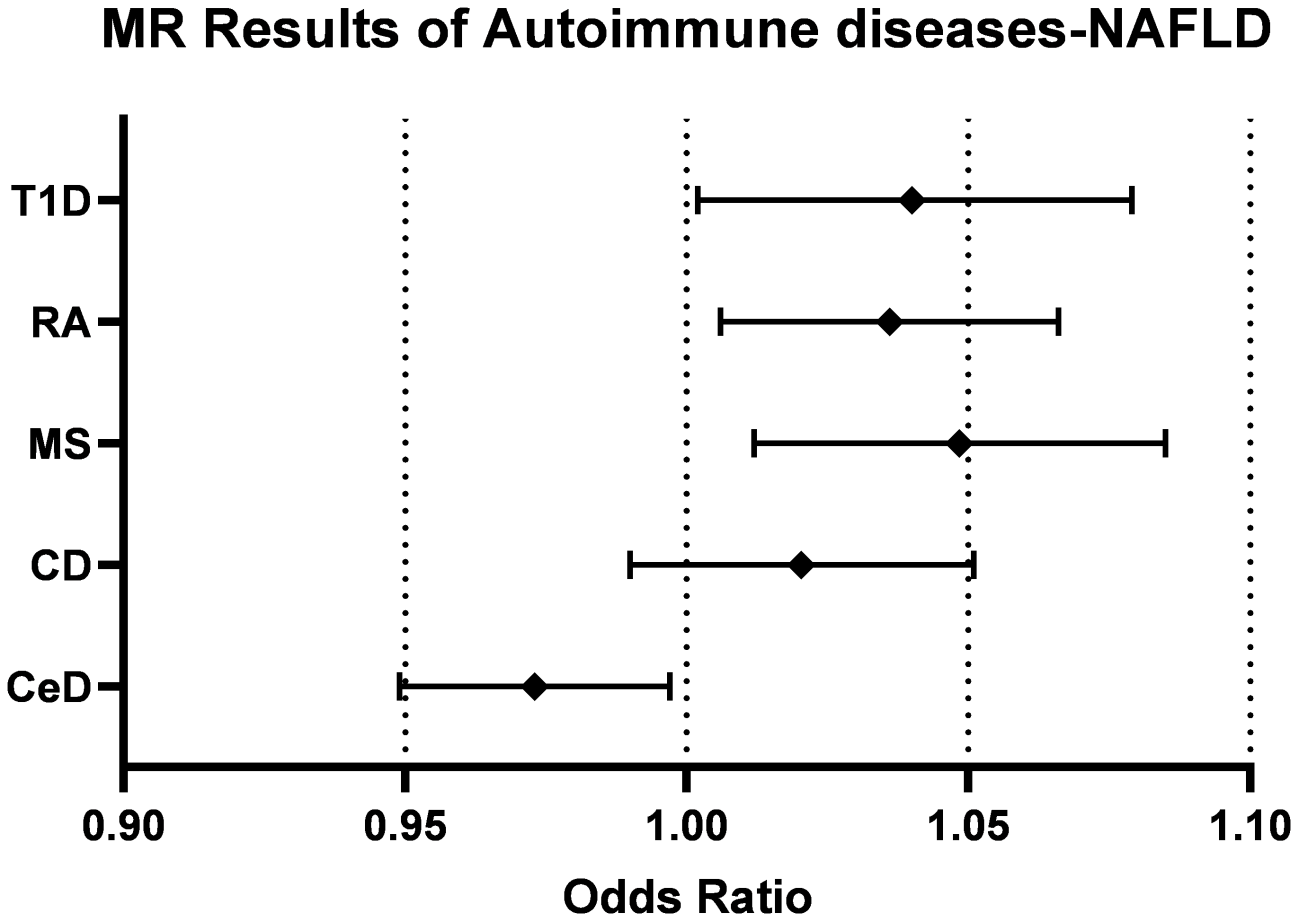
Forest plots of the causal effect of autoimmune diseases on NAFLD. CeD, celiac disease; CD, Crohn’s disease; MS, multiple sclerosis; RA, rheumatoid arthritis; T1D, Type 1 diabetes; NAFLD, non-alcoholic fatty liver disease.

Initially, neither CeD (OR=0.987, [0.971, 1.003], IVW p-value=0.118, FDR=0.131) nor CD (OR=1.027, [0.997,1.058], IVW p-value=0.082, FDR=0.103) was causally associated with NAFLD in the preliminary analysis. After adjustment for BMI and T2D, there was a significant negative association between CeD (OR= 0.973,[0.949,0.997], IVW p-value=0.026, FDR=0.065) and NAFLD, although there was still no causal association between CD (OR=1.020, [0.990, 1.051], IVW p-value= 0.185, FDR=0.185) and NAFLD (**Figure 2**). The results of other methods, such as IVW fixed effect model, weighted median method,and MR-Egger method, show consistent influence direction and significant association between CeD and NAFLD. (**Table 2**, **Supplementary Figures**) Cochran’s Q statistic (Q=24.508, p=0.789) suggested low heterogeneity, MR-Egger regression showed non-horizontal pleiotropy (intercept=0.011, p=0.061) and MR-PRESSO method did not find any outlier SNPs. (**Table 2**) Crohn’s disease was also less probable to be influenced by heterogeneity and horizontal pleiotropy (Q=113.068, p= 0.213; intercept=0.006, p=0.279) (**Table 2**, **Supplementary Figures**).

**Table 2:** MR Results of main methods.

Exposure	nSNP	IVW		IVW-fixed effect		Weighted median		MR-Egger		Simple mode		Weighted mode		P-heterogeneity	P-pleiotropy
		OR[95%LCI,95%UCI]	P	OR[95%LCI,95%UCI]	P	OR[95%LCI,95%UCI]	P	OR[95%LCI,95%UCI]	P	OR[95%LCI,95%UCI]	P	OR[95%LCI,95%UCI]	P		
Celiac disease	35	0.987[0.971,1.003]	0.118	0.987[0.970,1.004]	0.133	1.006[0.980,1.034]	0.635	0.980[0.958,1.003]	0.098	0.990[0.932,1.051]	0.735	0.998[0.974,1.022]	0.853	0.595	0.384
*Celiac disease	32	0.973[0.949,0.997]	0.026	0.973[0.949,0.997]	0.026	0.962[0.930,0.996]	0.026	0.944[0.909,0.981]	0.007	0.990[0.916,1.069]	0.791	0.953[0.924,0.983]	0.004	0.789	0.061
Crohn’s disease	115	1.027[0.997,1.058]	0.082	1.027[0.999,1.055]	0.054	1.012[0.971,1.054]	0.576	0.980[0.903,1.064]	0.634	1.027[0.932,1.132]	0.591	0.987[0.926,1.052]	0.693	0.053	0.234
*Crohn’s disease	103	1.020[0.990,1.051]	0.185	1.020[0.992,1.050]	0.163	1.007[0.963,1.053]	0.763	0.979[0.904,1.061]	0.608	1.024[0.924,1.134]	0.652	0.989[0.931,1.052]	0.735	0.213	0.279
Multiple sclerosis	44	1.047[1.012,1.083]	0.008	1.047[1.007,1.089]	0.022	1.037[0.979,1.098]	0.219	1.038[0.915,1.176]	0.569	1.068[0.959,1.190]	0.238	1.025[0.942,1.114]	0.575	0.888	0.884
*Multiple sclerosis	43	1.048[1.012,1.085]	0.008	1.048[1.007,1.090]	0.020	1.038[0.982,1.096]	0.187	1.038[0.915,1.177]	0.565	1.073[0.962,1.197]	0.211	1.029[0.945,1.121]	0.508	0.868	0.874
Rheumatoid arthritis	10	1.031[1.002,1.061]	0.034	1.031[1.002,1.061]	0.037	1.033[0.997,1.069]	0.071	1.005[0.957,1.055]	0.853	1.032[0.976,1.090]	0.298	1.032[0.995,1.072]	0.129	0.460	0.234
*Rheumatoid arthritis	9	1.036[1.006,1.066]	0.019	1.036[1.005,1.067]	0.022	1.035[0.998,1.073]	0.065	1.009[0.960,1.060]	0.734	1.045[0.991,1.103]	0.146	1.036[1.000,1.071]	0.089	0.471	0.235
Type 1 diabetes	27	1.038[1.001,1.076]	0.046	1.038[1.003,1.074]	0.036	1.059[1.004,1.118]	0.036	1.030[0.951,1.115]	0.470	1.060[0.963,1.168]	0.246	1.060[0.994,1.130]	0.085	0.322	0.844
*Type 1 diabetes	26	1.039[1.002,1.079]	0.041	1.039[1.004,1.076]	0.029	1.060[1.006,1.116]	0.029	1.032[0.952,1.118]	0.457	1.063[0.965,1.172]	0.225	1.062[0.995,1.133]	0.082	0.293	0.837

*means dataset after adjusting confounders.

The impact of genetic predisposition of MS (OR=1.047, [1.012,1.083], IVW p-value=0.008, FDR=0.040), RA (OR=1.031, [1.002,1.061], IVW p-value=0.034, FDR=0.066) and T1D (OR=1.038, [1.001,1.076], IVW p-value=0.046, FDR=0.066) all elevated the risk of NAFLD in IVW model. (**Table 2**) These conclusions were still valid after adjusting confounders, including MS (OR= 1.048, [1.012,1.085], IVW p-value= 0.008, FDR=0.040), RA (OR=1.036, [1.006,1.066], IVW p-value=0.019, FDR=0.063), T1D (OR= 1.039, [1.002,1.079], IVW p-value= 0.041, 0.066). (**Table 2**, **Figure 2**).

Sensitivity analysis indicated that the results were not biased by heterogeneity or horizontal pleiotropy: MS (Q=31.997, p=0.868; intercept=0.001, p=0.874), RA (Q=7.620, p=0.471; intercept=0.016, p=0.235), T1D (Q=28.322, p=0.293; intercept=0.002, p=0.837), MR-PRESSO method did not find any outlier SNPs (**Table 2**, **Supplementary Figures**).

IVW method indicated causal effect between genetically predicted BMI and NAFLD (OR= 1.715, [1.520,1.934], p-value= 1.701e-18). Genetically predicted T2D-NAFLD also revealed significant association in IVW method (OR= 1.104, [1.009,1.209], p-value= 0.032).

## 4 Discussion

Genetic susceptibility to all four diseases suggested a significant association with NAFLD, except for CD, which was not associated with NAFLD. CeD was negatively correlated with NAFLD. On the contrary, MS, RA, and T1D all increased the incidence of NAFLD slightly.

Celiac disease, an autoimmune disorder, predominantly impacts the small intestine and arises from the consumption of gluten in genetically predisposed individuals. CeD is widely believed to elevate the risk of NAFLD (7, 30). A cohort study indicated a 13.3 (95% CI 3.5-50.3) elevated risk of NAFLD in CeD patients compared to the general population in the first year after diagnosis of CeD, and continued to be significantly higher 15-year after diagnosis (HR = 2.5; 95% CI 1.0-5.9) (7). Another prospective cohort study showed that CeD patients with gluten-free diet(GFD) were at significantly increased risk of developing NAFLD (31), and that proton pump inhibitors and initial HOMA-IR may be risk factors for developing hepatic steatosis in this group of patients (32).

Previous studies found that CeD patients had a lower BMI than the general population at the time of diagnosis (33), and that this population was at a lower risk of metabolic syndromes such as T2D and hyperlipidemia before GFD treatment was administered (34–36). This may be related to the disruption of the intestinal barrier and relevant malabsorption induced by gluten consumption in CeD patients. NAFLD, on the other hand, is regarded as a fundamental hepatic manifestation of the metabolic syndrome (6), and The disease progression is associated with various risk factors, including obesity, hyperlipidemia, IR, and T2D (37).

The pathogenesis of CeD (38) and NAFLD (39, 40) are both correlated with gut microbiota. The spectrum of intestinal bacteria presented by the two diseases is seldom the same (41, 42), so we guess that the effects of intestinal flora disorders on organism metabolism are different tendencies in these two diseases. Most of the coexisting NAFLD in patients with CeD appear after GFD treatment (7), so the observed coexistence of CeD and NAFLD outcomes may be attributed to GFD treatment after CeD (7, 43). In recent years probiotics and other medications have also started to be utilized in most patients with intestinal diseases, and intestinal probiotic medications have also shown benefit in hepatic steatosis, the overall changes brought about by this new treatment still need further observed. In conclusion, the mechanisms of interaction between the two diseases still need to be further explored.

Our results did not show a causal association in CD-NAFLD, which is different from what we expected, but a previous MR study had similar findings (20). The risk of metabolic disease is lower in patients with CeD, which is the risk factor of NAFLD. The risk of metabolic disease is also low in IBD patients (44), but similar findings were not obtained in the causal association analysis of CD-NAFLD, either with or without adjustment for obesity and T2D. In clinical data, the prevalence of NAFLD in IBD patients varies 14.2%- 48% (8, 9, 45–47), and most studies had significantly higher prevalence compared to healthy controls, and we hypothesize that the treatment of IBD somehow influences the occurrence of subsequent NAFLD.

Most studies have shown that in IBD population, obesity, and diabetes remain risk factors for the development of NAFLD (9, 44, 46, 48), and a history of bowel resection, treatment of IBD with glucocorticoids, methotrexate, and azathioprine are associated with an increased incidence of NAFLD (8, 9, 44, 46), while the results of the anti-tnf-α therapy in patients with NAFLD are inconsistent (44, 49). In addition to known metabolic risk factors, treatment of IBD with glucocorticoids, azathioprine, methotrexate, and intestinal surgery seems to contribute to NAFLD. Notably, glucocorticoids and intestinal surgery may lead to hepatic steatosis or cholestasis (50), although without IBD. Therefore, we still cannot rule out a potential causal association of CD-NAFLD, and we still need to discuss the causal association of CD-NAFLD in larger sample-size GWAS studies and explore whether there are multiple factors whose pleiotropic effects counteract each other.

In our MR study, all three non-intestinal autoimmune diseases were associated with the presence of NAFLD, and the underlying mechanism of their interaction with NAFLD is presumed to remain closely related to the gut microbiota (51).

MS, idiopathic inflammatory demyelinating disease of the central nervous system (CNS), where damage to the blood-brain barrier is the initiating part of MS and is closely associated with subsequent demyelination, axonal injury, neuronal degeneration, and disease progression independent of relapse. Previous studies have indicated that gut microbiota can be involved in regulating the permeability of the blood-brain barrier, activating microglia expressing myelin-forming genes (52). The proportion of elevated intestinal permeability is significantly higher in MS patients than in healthy population (53), while previous studies have also suggested in animal models of MS that gut microbiota may regulate inflammatory cells migrate to the CNS by regulating blood-brain barrier integrity (54), thus we hypothesize that the MS-NAFLD association arises through the gut-brain axis and that molecular mimicry of gut microbiota may be a potential pathway of initiating autoimmune responses and the development of MS (55).

The mucosal origin hypothesis (56) suggested that the mucosa is the earliest site of RA-associated autoantibodies and that elevated levels of various types of RA-associated IgA in RA patients precede the onset of clinical arthritic symptoms (57, 58), while the homologous immune cells were found in the gut and joints of RA patients (59, 60), suggesting that gut-derived immunity in the preclinical phase is relevant to the pathogenesis of RA. The recent hypothesis of the gut-joint axis as a pathogenesis was then formulated, which mainly emphasizes the interaction between the mucosal immune system and the abnormal microbiota (61). Interestingly, gut dysbiosis has been shown to precede the onset of arthritis symptoms in both preclinical models and clinical samples and persist throughout the course of the disease (62), which seems to support a persistent role of gut microbiota in the disease.

T1D is characterized by pancreatic β-cell destruction leading to hyperglycemia and lifelong insulin reliance. The current view is that the presence of T1D exacerbates intrahepatic fat accumulation mainly through acquired insulin resistance (IR) (63). IR is a hit in the current multiple-hit theory of NAFLD (64), besides traditional factors such as sedentary, high-calorie diet, insulin dynamics in the portal circulation, genetic and epigenetic factors, and gut microbiota may contribute to hepatic and peripheral IR in T1D patients (65), leading to NAFLD.

Additionally, other factors may also contribute to NAFLD, such as oxidative stress, decreased physical activity, and drugs used such as corticosteroids. In NAFLD, intrahepatic lipid overload can activate multiple ROS-generating pathways leading to excessive production of oxidants, and high levels of ROS further affect oxidative modifications of cellular macromolecules (DNA, lipids, proteins, etc.), resulting in the accumulation of macromolecular damage and causing liver injury. Oxidative stress and lipid peroxidation are characteristic signs of NAFLD (66), while a common feature of autoimmune diseases such as MS, RA, and T1D is the increased production of ROS and RNS associated with the inflammatory response (67, 68). Oxidative stress can generate neoepitopes through oxidative modifications, and subsequently the organism produces autoantibodies corresponding to the neoepitopes (67), which could subsequently disrupt autoimmune tolerance. Furthermore, Motor system disorders resulting from different diseases such as MS and RA, which led to decreased physical activity, long-term use of corticosteroids in RA, and insulin use in T1D patients, can cause lipid metabolism disorders and weight gain in patient. Reduced physical activity itself is an important risk factor for NAFLD, and as mentioned above corticosteroids themselves may cause hepatic steatosis. Weight gain due to long-term insulin medication and IR itself are also involved in the development of NAFLD. Thus, the role of these factors either cannot be ignored.

The main strengths of our study include the elimination of potential confounders and reverse causal associations by MR analysis and control for heterogeneity and horizontal pleiotropy in the analysis. The study fulfilled the three major assumptions of MR, and the GWAS data related to outcomes were validated through known causal associations. However, there are several limitations: Firstly, all patients included in the GWAS data were Europeans, so it is possible that the results are not available for other ancestors, which means that our results need to be interpreted cautiously. Second, the data of outcomes are aggregated from several different sources and cases all identified through EHRs (electronic health records) (17), so the possibility of misclassification of NAFLD cannot be excluded, even if we validated the summary data. Furthermore, various statistical methods cannot exclude horizontal pleiotropy induced by biological mechanisms, so we should still be concerned about the impact of horizontal pleiotropy on study results, especially null causal association results. Finally, only several autoimmune diseases were analysed in this study due to the lack of support from epidemiological studies of diseases such as SLE and GD with NAFLD.

## 5 Conclusion

In conclusion, there is a considerable association between gene-driven autoimmune diseases and NAFLD, with CeD negatively associated with NAFLD and MS, RA, T1D positively associated with NAFLD. This result suggests that CeD itself may be a beneficial for NAFLD, suggesting that previous observational studies may be influenced by confounding factors, and patients with MS, RA, and T1D may have slightly higher morbidity of NAFLD than the healthy population, monitoring the prevalence of NAFLD in these populations is considerable. In addition, causal association between CD and NAFLD was ruled out.

## Data availability statement

Publicly available datasets were analyzed in this study. This data can be found here: ebi-a-GCST005523, ieu-a-12, ebi-a-GCST005531, ieu-a-834, ebi-a-GCST010681, GCST90091033.

## Ethics statement

The current study was based on summary-level GWAS data and each GWAS was approved by its corresponding ethical committee. All datasets can be downloaded freely without restriction.

## Author contributions

MX, and TW contributed equally to this manuscript. All authors contributed to the article and approved the submitted version.

## Supplementary material

Supplementary Figures Scatterplots of the causal effect of autoimmune diseases (**A**: CeD, **B**: MS, **C**: RA, **D**: T1D as exposure) on NAFLD in adjusted MR. Analyses were conducted by IVW, IVW-fixed effects, weighted median, MR-Egger, Weighted mode and Simple mode methods. IVW, inverse variance weighted; MR, Mendelian randomization; MR-Egger, MR-Egger regression; NAFLD, non-alcoholic fatty liver disease.
