# Supplementary material for: Influence of genetically predicted autoimmune diseases on NAFLD
Source: Front Immunol. 2023 Sep 11;14:1229570. doi: 10.3389/fimmu.2023.1229570 (PMC10520707; doi:10.3389/fimmu.2023.1229570)
Supplement: Supplementary Figures — Scatterplots of the causal effect of autoimmune diseases (A: CeD, B: MS, C: RA, D: T1D as exposure) on NAFLD in adjusted MR. Analyses were conducted by IVW, IVW-fixed effects, weighted median, MR-Egger, Weighted mode and Simple mode methods. IVW, inverse variance weighted; MR, Mendelian randomization; MR-Egger, MR-Egger regression; NAFLD, non-alcoholic fatty liver disease. [file DataSheet_1.pdf]

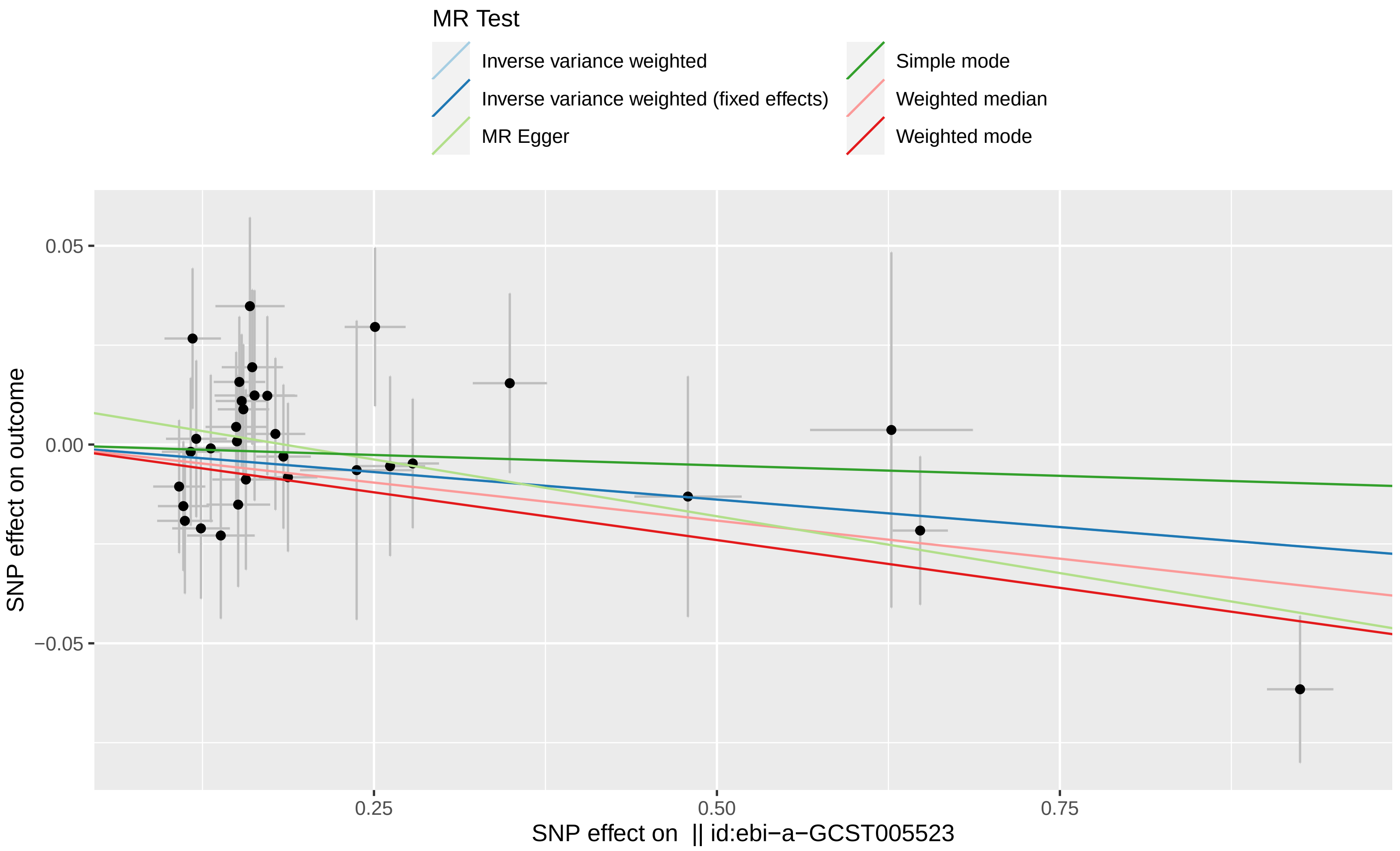
MR Test
Inverse variance weighted
Inverse variance weighted (fixed effects)
MR Egger
Simple mode
Weighted median
Weighted mode
SNP effect on outcome
0.05
0.00
−0.05
0.25
0.50
0.75
SNP effect on || id:ebi−a−GCST005523
