# Supplementary figures and images for: Influence of genetically predicted autoimmune diseases on NAFLD
Source: Front Immunol. 2023 Sep 11;14:1229570. doi: 10.3389/fimmu.2023.1229570 (PMC10520707; doi:10.3389/fimmu.2023.1229570)

# MR Test

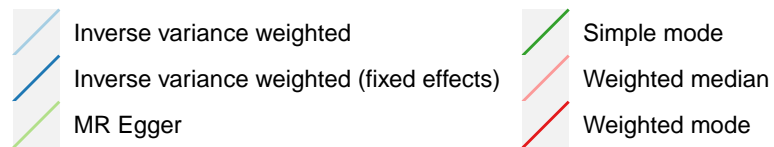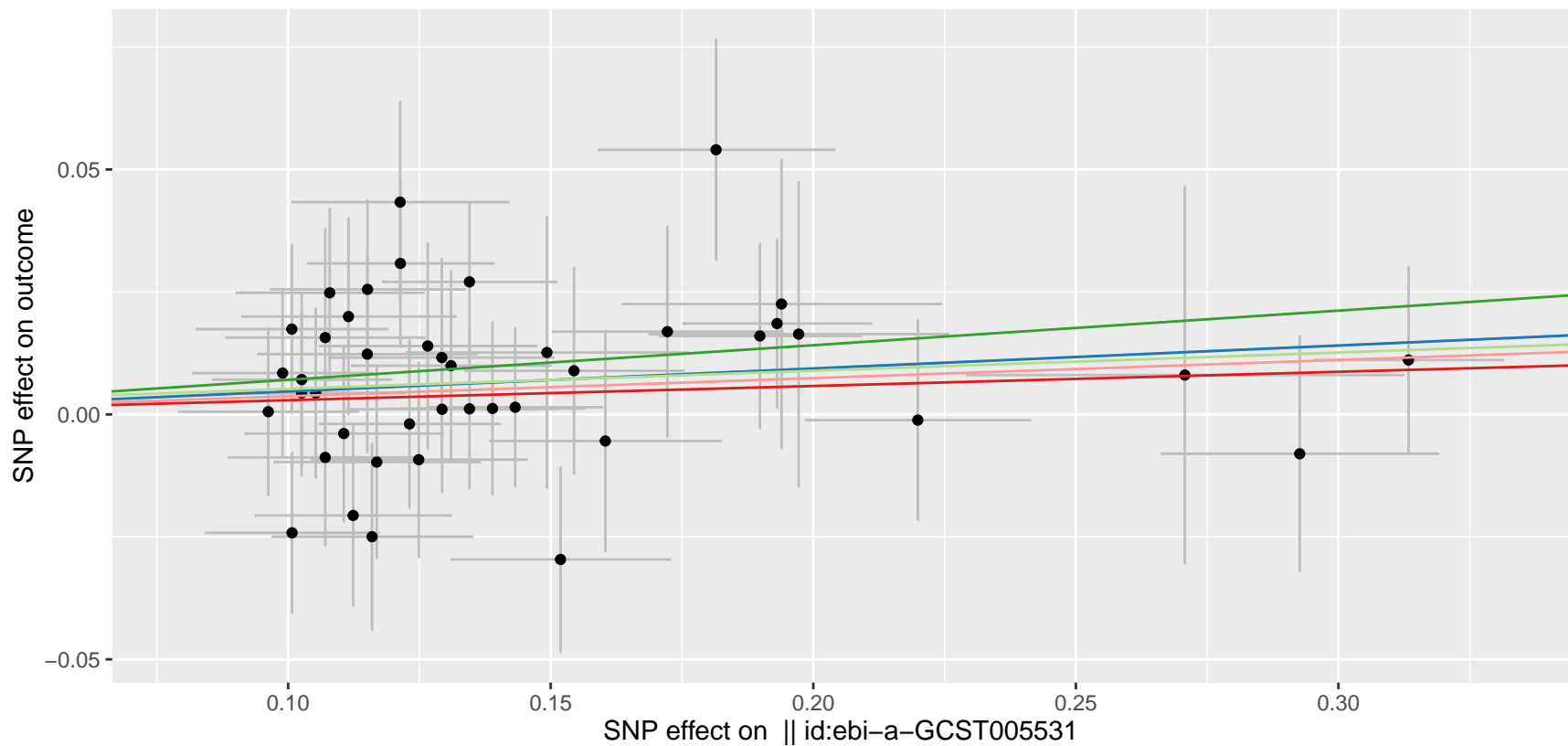

Supplement: Supplementary file 2 [file DataSheet_2.pdf]

# MR Test

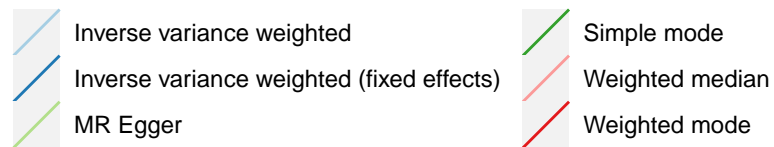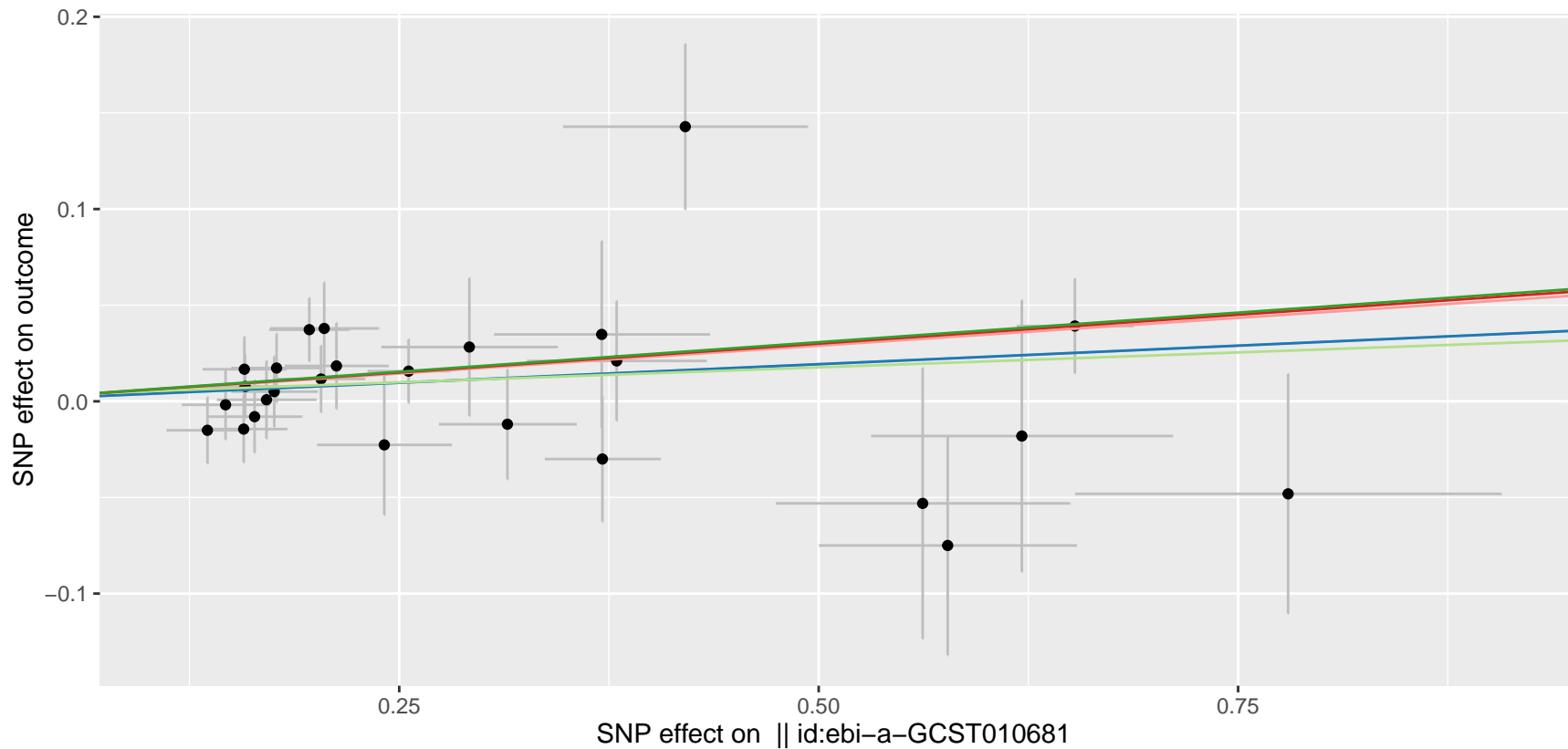

Supplement: Supplementary file 4 [file DataSheet_4.pdf]
