# Supplementary material for: Influence of genetically predicted autoimmune diseases on NAFLD
Source: Front Immunol. 2023 Sep 11;14:1229570. doi: 10.3389/fimmu.2023.1229570 (PMC10520707; doi:10.3389/fimmu.2023.1229570)
Supplement: Supplementary file 3 [file DataSheet_3.pdf]

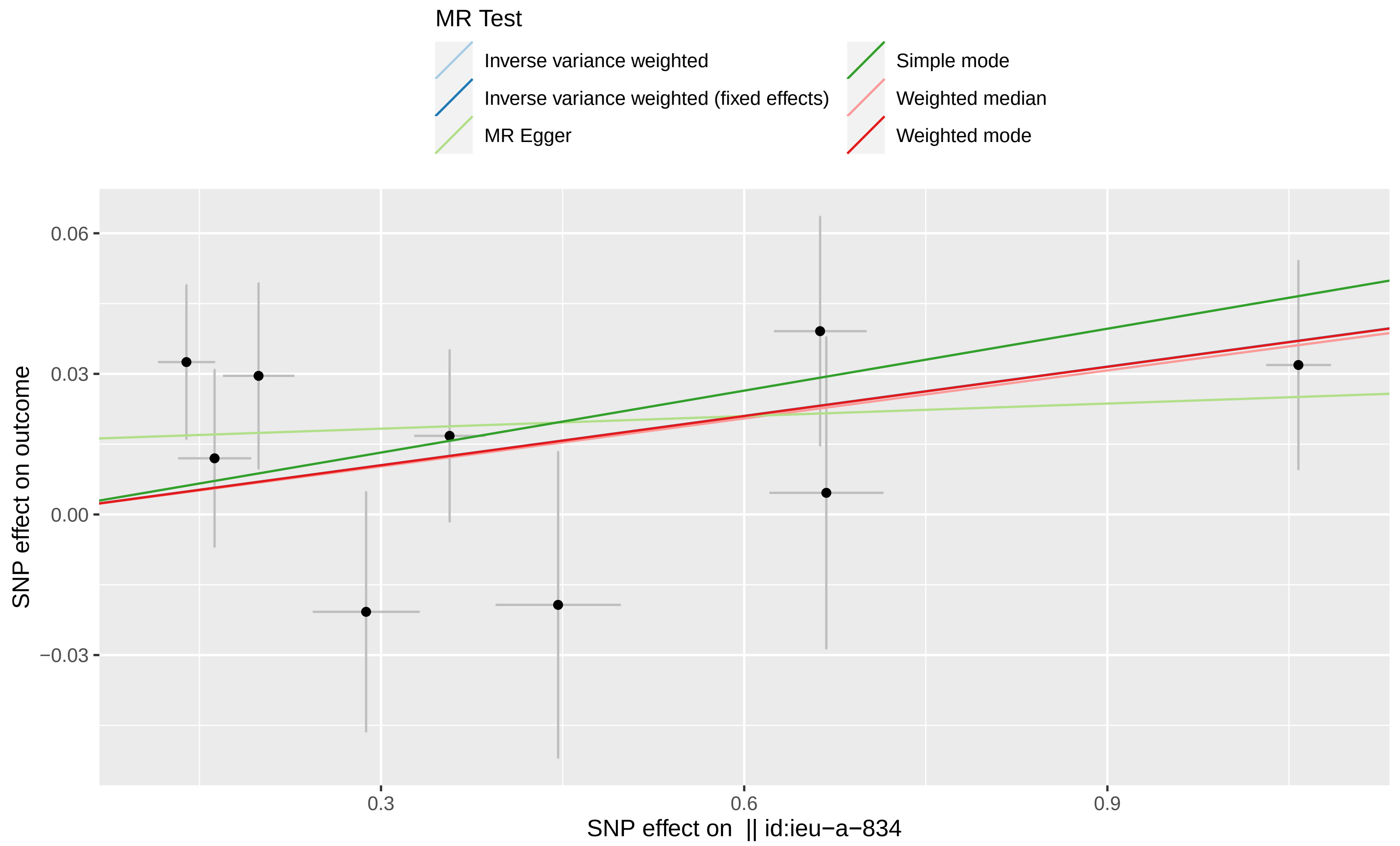
MR Test
Inverse variance weighted
Inverse variance weighted (fixed effects)
MR Egger
Simple mode
Weighted median
Weighted mode
0.06
0.03
0.00
−0.03
SNP effect on outcome
0.3
0.6
0.9
SNP effect on || id:ieu−a−834
